# Choline Binding Proteins from *Streptococcus pneumoniae*: A Dual Role as Enzybiotics and Targets for the Design of New Antimicrobials

**DOI:** 10.3390/antibiotics5020021

**Published:** 2016-06-14

**Authors:** Beatriz Maestro, Jesús M. Sanz

**Affiliations:** Instituto de Biología Molecular y Celular, Universidad Miguel Hernández. Av. Universidad s/n, Elche 03202, Spain

**Keywords:** pneumococcus, choline-binding protein, endolysin, enzybiotic, cell-wall hydrolase, adhesin

## Abstract

*Streptococcus pneumoniae* (pneumococcus) is an important pathogen responsible for acute invasive and non-invasive infections such as meningitis, sepsis and otitis media, being the major cause of community-acquired pneumonia. The fight against pneumococcus is currently hampered both by insufficient vaccine coverage and by rising antimicrobial resistances to traditional antibiotics, making necessary the research on novel targets. Choline binding proteins (CBPs) are a family of polypeptides found in pneumococcus and related species, as well as in some of their associated bacteriophages. They are characterized by a structural organization in two modules: a functional module (FM), and a choline-binding module (CBM) that anchors the protein to the choline residues present in the cell wall through non-covalent interactions. Pneumococcal CBPs include cell wall hydrolases, adhesins and other virulence factors, all playing relevant physiological roles for bacterial viability and virulence. Moreover, many pneumococcal phages also make use of hydrolytic CBPs to fulfill their infectivity cycle. Consequently, CBPs may play a dual role for the development of novel antipneumococcal drugs, both as targets for inhibitors of their binding to the cell wall and as active cell lytic agents (enzybiotics). In this article, we review the current state of knowledge about host- and phage-encoded pneumococcal CBPs, with a special focus on structural issues, together with their perspectives for effective anti-infectious treatments.

## 1. Introduction

*Streptococcus pneumoniae* (the pneumococcus) is a Gram-positive bacteria, responsible for acute life-threatening infections including pneumonia, meningitis and sepsis [1], and constitutes the most frequently detected pathogen in cases of community-acquired pneumonia [2]. It is known that bacterial pneumonia is the major cause of childhood mortality worldwide along with malnutrition, so it has been labeled as “The forgotten killer of children” by the United Nations Children’s Fund (UNICEF) and the World Health Organization (WHO) [3]. Besides, it is a major causative agent of otitis media [4]. Pneumococcal diseases are widespread both in developed and developing countries, leading to more than 1.6 million deaths per year according to WHO [5], half of them in children under five and accounting for about 11% of all childhood deaths worldwide [1]. Pneumococci are commonly found asymptomatically in the upper respiratory tract of around half of the infant population, providing a natural reservoir and supplying a mechanism for person to person transmission [6]. Differences in the immunochemistry of the polysaccharide capsule has led so far to the identification of 94 serological types, of which only between 30 and 40 have been unequivocally associated with pneumococcal disease [7].

Current antipneumococcal strategies are aimed towards vaccination and antibiotic treatment. The widespread implementation of the 7-valent pneumococcal conjugate vaccine (PCV7, Wyeth/Pfizer, Prevnar^®^) in children led to a dramatic reduction in PCV7-type invasive disease and carriage, not only in vaccinated children but also in unvaccinated persons of all ages [8,9,10]. Two higher valency PVC vaccines have been introduced in recent years: the 10-valent (PCV10, GSK, Synflorix^®^), including the seven serotypes of PCV7 plus serotypes 1, 5, and 7F, and the 13-valent (PCV13, Wyeth/Pfizer, Prevnar13^®^), containing the PCV10 serotypes plus additional 3, 6A, and 19A serotypes, the last one being the only one of the three licensed for use in adults over 50 years of age [11,12,13,14]. Despite these efforts, present-day vaccination strategies face several drawbacks. For instance, serotype replacement phenomena have been reported [15,16] so that non-vaccine serotypes may come to dominate in the mid-term, causing reemergence of disease. Moreover, vaccines do not always protect from invasive pneumococcal disease in developing countries, either because serotypes other than those in developed countries are predominant, or because of insufficient access of the population to vaccination programmes [17].

Regarding antibiotic therapy, the so-called “antibiotic era of drug discovery” (1920s–1960s) witnessed the appearance of a number of molecular classes that constitute the basis of most of the antimicrobials in use today. However, discovery of fundamentally new classes of antibiotics came to an almost complete halt after the mid-1960s [18] despite the fact that the proportion of antibiotic resistant bacteria had been increasing over this period. Antimicrobial resistance (AMR) has indeed impacted on the prevalence of *S. pneumoniae.* Since 1967, the incidence of pneumococcal AMR has been steadily increasing and resistance to β-lactam antibiotics is now widespread [19]. *S. pneumoniae* isolates not susceptible to penicillin amounted to 35% in 2004 in the U.S., whereas data from Europe varies significantly between countries reached levels of up to 50% in some Southern [20,21] regions, reflecting the degree of exposure of the individual to non-controlled antibiotic administration [19]. Moreover, the number of cases due to strains that are not susceptible to fluoroquinolones and macrolides is also increasing [19,20,22].

From the above situation, it is evident that investigations of potential new drug targets in *S. pneumoniae* should include virulence factors common to all pneumococcal serotypes, which may represent targets for effective and selective chemotherapy and may circumvent therapeutic problems due to drug resistance. Such novel targets may be found in the bacterial cell wall, a traditional and excellent source for the development of new antibiotics [23].

**Figure 1:**
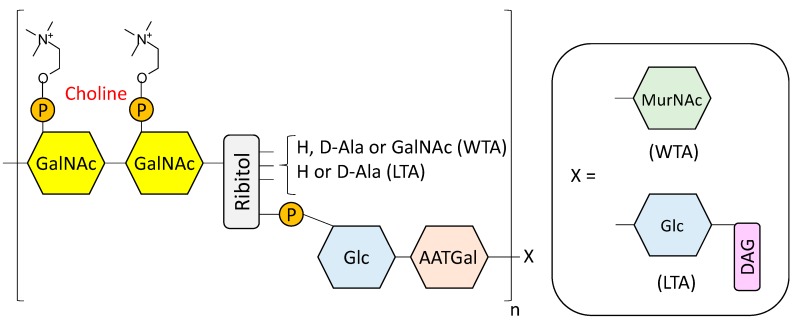
Structural model of pneumococcal cell wall teichoic (WTA) and lipoteichoic (LTA) acids according to Seo *et al.* [26] and subsequently refined by Gisch *et al.* [27]. GalNAc, *N*-acetylgalactosamine; P, phosphate group; Glc, glucose; AATGal, 2-acetamido-4-amino-2,4,6-trideoxygalactose; MurNAc, *N*-acetylmuramic acid; DAG, diacylglycerol.

The pneumococcal cell wall is a complex structure composed of multiple layers of peptidoglycan (PG, also known as murein) containing units of teichoic acid (TA) that is covalently bound to the wall (WTA, also known as C-polysacharide) or to the membrane (lipoteichoic acid, (LTA, also referred to as F-antigen) [24,25,26,27,28]. The PG structure consists on alternating units of *N*-acetylglucosamine (NAG) and *N*-acetylmuramic acid (NAM) linked by β(1,4) bonds [29]. Short peptide chains of three to five amino acid residues are bound to distant NAM residues, knitting a crosslinked mesh [29,30]. On the other hand, the basic repeating unit for TA is comprised of 2-acetamido-4-amino-2,4,6-trideoxy-d-galactose, d-glucose, ribitol-phosphate and two residues of *N*-acetylgalactosamine (GalNAc) [26,31,32,33] (Figure 1), whereas glucose is replaced by galactose in the TA of a serotype 5 strain [34]. Either one or both of the GalNAc residues (depending on the strain) are modified with phosphorylcholine (P-Cho) [31,35,36], an amino alcohol that can also be found in the surface of several other microorganisms [37,38,39,40], including pathogens that reside primarily in the mammalian airway [41].

The P-Cho groups present in the TA of the pneumococcal envelope are involved in several physiological functions [42,43]. They are recognized by the C-reactive protein (CRP) [44] and l-ficolin [45], both immune-system elements that induce complement activation, leading to host protection against infection. Moreover, attachment of bacterial P-Cho to the G-protein-coupled platelet-activating factor (PAF) receptor promotes adherence to the host cell [46], so that pneumococcal strains lacking P-Cho are unable to colonize the upper respiratory tract in mice and are less virulent in a murine sepsis model [47]. Finally, choline moieties present on the cell wall constitute specific attachment ligands for surface proteins, and also represent an anchor for bacteriophages [48].

*S. pneumoniae* exhibits a unique auxotrophic requirement for choline [49,50], although some choline-independent pneumococcal mutants have been isolated [51,52,53]. Pneumococci are also able to grow in the presence of choline analogues such as ethanolamine that are incorporated in the TA, although they cannot replace the P-Cho functions [54,55], as in these conditions the bacteria cannot undergo genetic transformation nor autolysis, are resistant to bacteriophage, and form long chains [54]. All these properties are also attributable to pneumococcal choline-independent strains [51,52,53].

The pneumococcal cell wall contains a variety of surface proteins different from those involved in cell-wall synthesis and turnover, like the so-called choline-binding proteins (CBPs) [37,38,42,48,56,57]. These polypeptides are found in all pneumococcal strains, and their number varies from 13 to 16. They take part in central metabolic processes, and their classification is sometimes confusing as some of them have received different names despite corresponding to the same gene [38,42,58]. Included in this group there are murein hydrolases, which have a key role in remodeling the cell wall in crucial steps for the bacterial growth and division, as well as host-cell adhesins and other virulence factors. Furthermore, the CBP family also includes several bacteriophage-encoded lytic enzymes.

**Figure 2:**
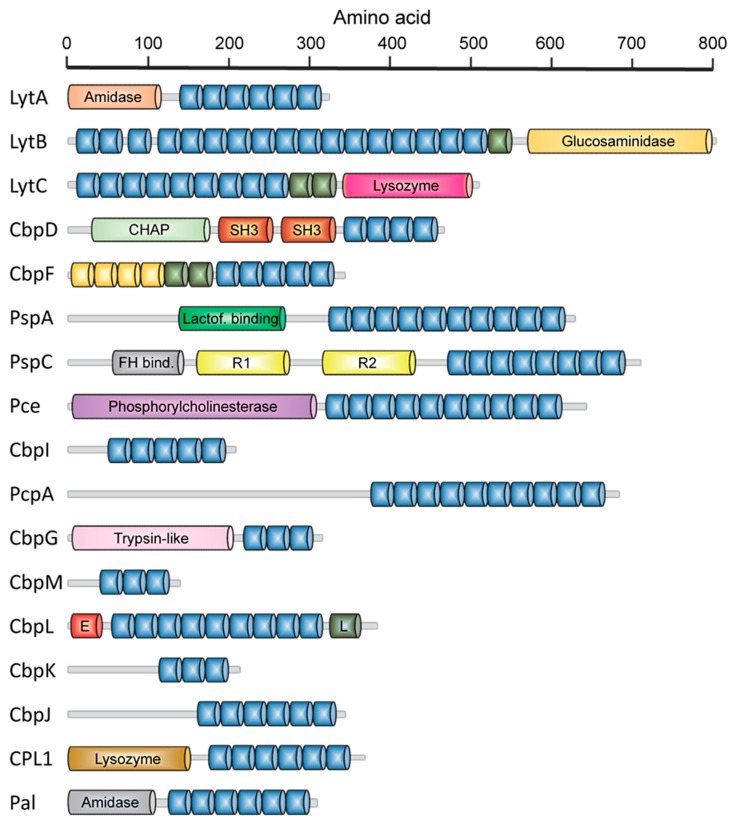
Modular organization of host- and phage-encoded pneumococcal CBPs. Blue units represent the standard choline-binding repeats (CBRs) which configure the choline-binding modules (CBM). Non-consensus CBRs are shown in green. Yellow units represent repeats belonging to the *CW_binding_1* PFAM family but unable to bind choline. Activities ascribed to several functional modules are shown: *CHAP*, cysteine, histidine-dependent amidohydrolase/peptidase module; *Lactof. binding*, lactoferrin-binding domain; *FH bind*., factor H-binding domain; *R1* and *R2*, domains with adhesin functions; *E*, “Excalibur” domain; L, “Lipoprot” domain.

**Figure 3:**
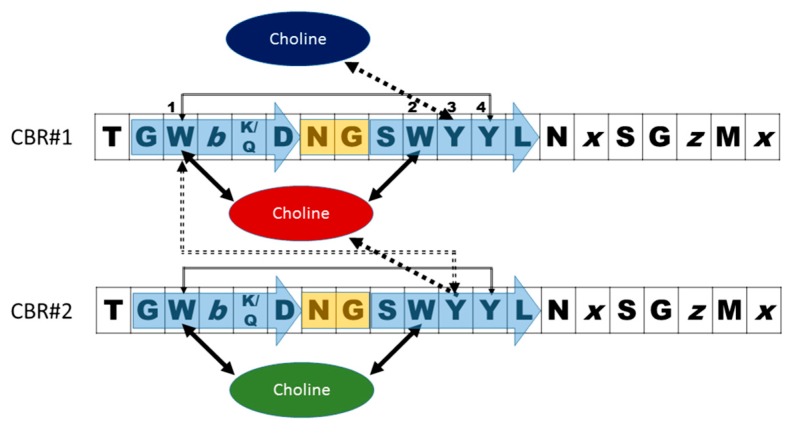
Scheme of a standard pneumococcal choline-binding site configured by two consecutive CBRs with the consensus sequence deduced by Jalview [71,72]. *b* is a hydrophobic residue, *z* has a small side chain and *x* may be any residue. The blue arrows and orange rectangle indicate the strand-turn-strand configuration of the β-hairpin. Black, solid arrows show the cation-π interactions with choline. Grey arrows indicate stabilizing π-π stacking between aromatic side chains. Dashed arrows denote a contact that may be found either as cation-π or π-π types. Numbers indicate the situation of aromatic residues in the repeat.

**Figure 4:**
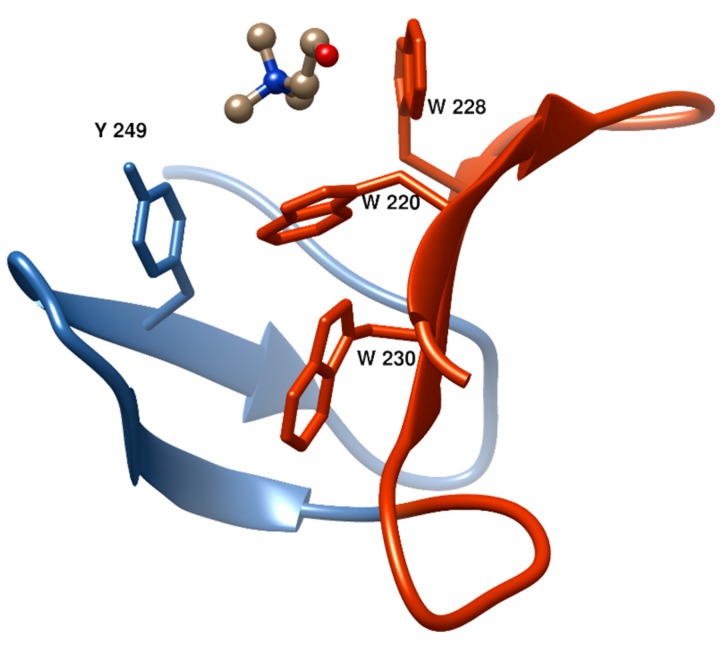
Structure of a typical choline-binding site in the LytA amidase [73] (PDB code; 1GVM] showing two consecutive CBRs (in **blue** and **dark orange**) and the aromatic residues involved in choline binding. This and the rest of figures showing 3D structures were prepared with UCSF Chimera 1.10 [74].

Early genetic and biochemical studies strongly suggested that CBPs could be the result of the fusion of two independent modules: a functional module (FM) and a highly conserved choline-binding module (CBM), which allows the non-covalent binding to P-Cho residues [59,60,61,62] (Figure 2). The CBM is usually located at the C-terminus (with the exception of LytB and LytC), and sometimes is preceded by a proline-rich linker sequence as in PspA and PspC [63,64]. In turn, CBMs are built up from the tandem concatenation of short, aromatic-rich units (choline binding repeats, CBRs) about 20 aa long (Figure 3). The CBRs belong to the widespread *CW_binding_1* motif family that can be found in many different organisms (PFAM code PF01473) [65]. The *CW_binding_1* family not only includes pneumococcal CBRs but also glucan-binding polypeptides from oral streptococci, *Leuconostoc* dextransucrases and clostridial toxins. The analysis of chimeric genes between phages and bacteria have led to postulate an evolutionary relationship from a common ancestral module [66,67,68,69,70] or duplication events from an ancestral motif [37] in which the CBRs are presented as ubiquitous sequences belonging to a more general “putative cell wall binding repeat”. Although some consensus sequences have been previously described for CBRs in the past, we have carried out an updated Jalview analysis [71,72] of the current content of *CW_binding_1* members, and focusing only on the specific subset of sequences of pneumococcal origin. Such analysis yields the consensus sequence **TGW-*b*-(K,Q)DNGSWYYLN-*x*-SG-*z*-M-*x*_1-2_** (positions amounting for more than 30% conservation are specified), where *b* is a hydrophobic residue, *z* has a small side chain and *x* is any residue. Structurally, the CBR acquires a β-hairpin conformation of approximately 14-aa followed by a 6-7-residue linker (Figure 3 and Figure 4). The β-hairpins of the CBRs are usually three-dimensionally arranged as β,β-3 solenoid superstructures (see below), configuring choline binding sites between two consecutive repeats.

The first and second aromatic residues in the repeat #1, usually tryptophans, are disposed in right angle and establish cation–π interactions with choline. (Figure 4). The third aromatic from the repeat #2, normally a tyrosine, is sometimes found establishing an additional cation-π interaction with the ligand, although in many occasions this interaction is lacking and, instead, this residue interacts with the first tryptophan of repeat #1 through a T-shaped π-π stacking, as it is shown in Figure 4. Remarkably, a fourth aromatic residue within the hairpin is invariably found establishing a T-shaped π-π stacking with the first aromatic side chain located at the bottom of the binding pocket, (Figure 3 and Figure 4). This interaction has been generally overlooked in the structural analyses of CBPs despite its highly stabilizing nature [75], which might be decisive to consolidate the rigid architecture of the site. The set of interactions is usually completed with the eventual participation of a hydrophobic side chain from the C-terminal part of the repeat. Very diverse choline-binding affinities have been described for different CBPs in spite of the conservation of the overall geometry of the standard binding sites among all members. Altogether, these data suggest that the interactions between choline in the cell wall and the choline-binding sites comprise the existence of a complex interplay of forces, especially of the π-π and cation-π type, very sensible to subtle differences [75]. Finally, despite their small size, CBRs behave as highly autonomous folding units, since the central β-hairpin of a single repeat is able to acquire its native structure in solution in the absence of the rest of the protein and to recognize choline and choline analogues, albeit with residual affinity [76,77].

The highly variable number of CBRs present in CBPs (Figure 2) together with stepwise deletion studies carried out on the CBM from the LytA amidase [78,79], that originate truncated proteins with diminished ligand recognition, led to the hypothesis that the affinity for choline is directly related to the number of CBRs in the protein. However, it should be pointed out that the mentioned deletions also prevent LytA dimerization, which is essential for full stability and activity (see below). Instead, LytC contains 7 choline-binding sites per monomer [80] whereas CPL1 only possesses 2 of them [81], and yet the affinities for free choline in solution are similar [82]. In the same vein, LytA contains 5 full and 1 half binding sites per monomer [83], and nevertheless it shows a lower affinity for choline than CPL1 with only 2 sites [82,84]. All these data seems to rule out that the mere increase in CBRs leads to stronger choline binding, at least in the ligand soluble form. However, it should be remarked that the cell wall is a multivalent substrate that adheres simultaneously to all choline-binding sites and that only systematic studies on binding to macromolecular supports mimicking the natural bacterial surface can yield a definitive answer to this question.

Alternative variants have also been described that differ from standard CBRs in peptide length and in the number of aromatic residues involved in choline binding (between 2 and 6) [38]. Especially interesting are those containing the GYMA subsequence, with 6 aromatics from three consecutive CBRs contributing to binding. The role of these non-canonical GYMA sites is unknown but they have been postulated to be responsible of increasing the affinity towards the TA units by additionally recognizing the *N*-acetylgalactosamine residues [80]. It is noteworthy that these GYMA sites have been described so far only in the two CBPs, LytC and LytB, that harbor the CBM at the N-terminal region [80]. The inclusion of non-canonical CBRs often lead to distortions in the solenoid arrangement and are crucial to regulate the activity of the protein either by promoting intramodular contacts or affecting (and even losing) the choline binding properties.

Despite its biological relevance, research on antipneumococcal therapies based on CBPs still constitutes an almost unexplored field. Several CBPs are being investigated for new vaccine formulations [64,85,86,87,88,89], whereas the lytic activity of phage- and host-lysins has proved to be very efficient for protection from pneumococcal infections. On the other hand, discovery of ligands specifically inhibiting CBPs displays several potential advantages, as CBPs are common to all serotypes and play essential roles in cell physiology and virulence. Furthermore, a single inhibitor might simultaneously affect the whole family for their attachment to the cell wall, thus hampering the appearance of resistances.

In this review, we analyze the most relevant characteristics of host- and phage-encoded pneumococcal CBPs, with a special focus in structure-function relationships, and assess their dual role as active therapeutic agents and targets for novel antimicrobials.

## 2. Pneumococcal Host-Encoded CBPs

### 2.1. LytA Amidase

LytA is an *N*-acetylmuramoyl l-alanine amidase (NAM-amidase) involved in the autohydrolysis of the cell wall at the end of the stationary phase in liquid cultures [90]. It is highly conserved among pneumococci, exhibiting only slight sequence diversity [91,92]. Its function *in vivo* is related to the virulence mediated by cellular lysis, possibly due to the discharge of other virulence factors such as pneumolysin [93,94], or by inducing the release of proteins that help evading the host immune response [95]. LytA autolysin is known to contribute to the lysis of pneumococci induced by antibiotics such as penicillin and vancomycin, as well as deoxycholate [96,97,98,99]. It also participates in the so-call “fratricide” phenomenon (see below). Interestingly, a new role for this enzyme has been recently reported as an active participant in capsule shedding, induced in response to the interaction with cationic antimicrobial peptides present in the human epithelium, and with the consequence of increasing the bacterial invasiveness [100].

LytA is mainly localized in the cytoplasm (~95%), and only a small fraction is found associated to the extracellular cell wall (~5%) [101]. The cells are protected from the autohydrolytic activity of LytA during logarithmic growth, whereas the extracellular fraction of LytA slowly increases to ~30% during the stationary phase, after which autolysis is induced resulting in a major release of the autolysin [101]. However, it is still unclear how LytA translocates from the cytoplasm, as it does not contain any recognizable signal peptide for protein secretion.

**Figure 5:**
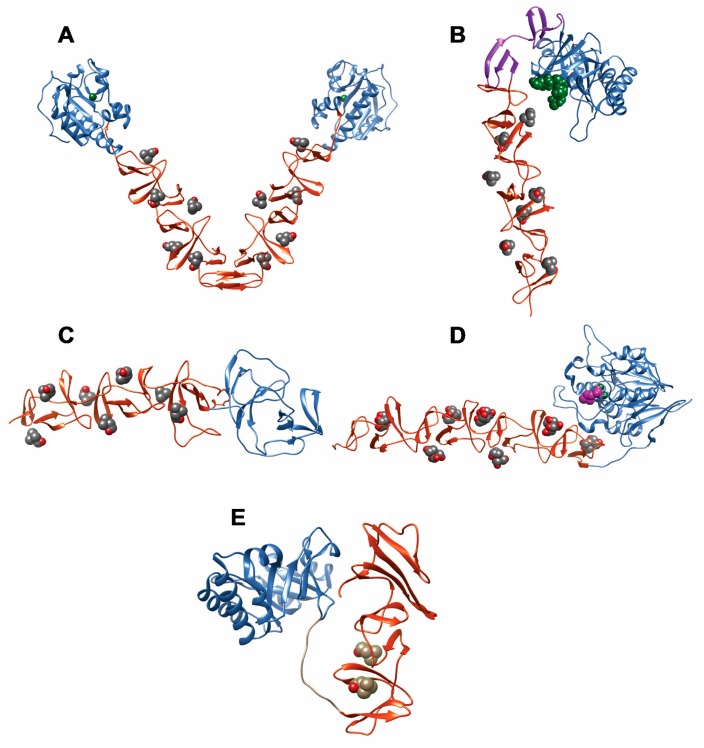
Three-dimensional structures of complete or nearly complete CBPs. The FM and CBM modules are colored blue and orange, respectively, and ligands are shown in van der Waals representation. (**a**) LytA amidase (PDB code: 4X36); the Zn^2+^ ion is colored green and the choline ligand is shown in CPK color scheme; (**b**) LytC lysozyme (PDB code: 2WWD); the NI and NII domains within the CBM are colored orange and magenta, respectively; choline is CPK colored; the PG fragment *N*-acetylmuramyl-l-alanyl-d-isoglutamine is shown in green; (**c**) CbpF protein (PDB code: 2V04); choline is CPK colored; (**d**) Pce cholinesterase withouth the C-terminal 85-aa tail (PDB code: 2BIB); phosphocholine bound in the active site is shown in magenta, and the Zn^2+^ ions are colored green; bis-tris molecules (in CPK scheme) occupy the choline-binding sites; (**e**) CPL1 lysozyme bound to choline (PDB code: 1OBA).

The three-dimensional structure of the entire LytA amidase has been recently solved [83] (PDB code: 4X36] and confirms the overall characteristics deduced from the previous analysis of the separate functional [102] (PDB code: 4IVV) and choline-binding modules [73,103,104] (PDB codes: 1HCX, 1H8G, 1GVM and 2BML). The protein acquires an elongated form with a globular amidase domain and an extended CBM with 6 CBRs plus a hairpin tail (Figure 5A). In the presence of choline, dimerization occurs through a C-terminal β-hairpin in the CBM, resulting in a “boomerang-like” shape that is presumably fitted to access to the peptidoglycan network, as the distance between amidase modules is similar to that predicted between lactyl-amide N atoms [83].

The amidase module folds with a central seven-stranded β-sheet and six flanking α-helices forming a wide bifurcated binding cavity with a catalytic Zn^2+^-containing center located at the bottom. This groove has been proposed to accommodate the glycan chain and the peptide stem of nascent PG [83,102].

The folding and stability of the CBM from LytA has been thoroughly studied. The module folds as an elongated left-handed β,β-3-solenoid formed by the stacking of 6 β-hairpin structures connected by loops and corresponding to the CBRs [73,83,103,104] (Figure 5A). Six choline binding sites are formed between consecutive CBRs, all of them following the standard binding mode described above with the exception of a half-site provided by the N-terminal CBR which binds choline only with residual affinity [83]. Calorimetric and spectroscopic analyses have demonstrated a complex choline binding mechanism that involves the presence of low-affinity and high-affinity choline-binding sites [84,105,106]. Binding of choline promotes dimerization of the protein through a C-terminal, non-CBR hairpin tail (Figure 5A) and confers stability against chemical and thermal denaturation [84,105,106], probably by shielding the otherwise solvent-exposed hydrophobic patches between CBRs [73].

**Figure 6:**
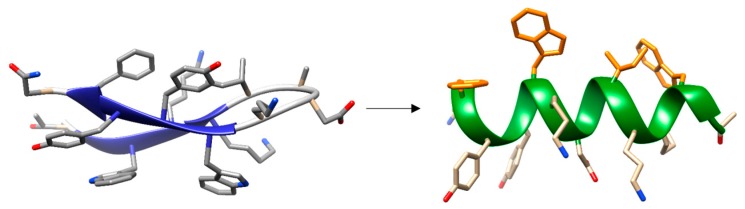
Micelle-induced conformational change of the isolated β-hairpin corresponding to the LytA 239-252 sequence. Non-polar residues in one face of the α-helical structure are colored orange to show the amphipathycity of the helix.

LytA dimerization is essential for an efficient activity of the protein. Truncated [78,79] and point-mutated [107] forms of LytA involving the C-terminal, dimerization hairpin, result in severe loss of stability and enzymatic activity, pointing to Ile-315 as a key amino acid residue in both enzymatic activity and folding [107]. Besides, the study of the equilibrium unfolding of the 194–318 sequence from the LYTA (C-LytA protein), lacking the first CBR, has unveiled the accumulation of partly folded intermediates and a relevant structural independence between hairpins, especially in the absence of choline [79,106,108]. The complexity of the CBM from LytA is also revealed in two additional aspects. First, the protein has been demonstrated to be synthetized in the cytoplasm as an inactive, “E-form” that can be “converted” to the active “C-form” upon incubation with choline or cell wall preparations at low temperature [109]. The molecular basis of this mechanism is still unresolved [107]. Moreover, a recent study shows that both C-LytA and individual CBRs have the capacity to undergo reversible disruption of their native β-hairpin structure to acquire an alternative, α-helical conformation with the ability to recognize detergent micelles and lipid vesicles [77] (Figure 6). This unusual conformational change might fulfill a physiological role, such as the help to complete the sorting of this protein to the bacterial surface through a signal peptide-independent mechanism.

### 2.2. LytB N-Acetylglucosaminidase

The pneumococcal endo-β-*N*-acetylglucosaminidase LytB is a non lytic enzyme that plays a critical role in cell separation during division [110,111] with the assistance of LytA [112]. LytB is specifically located at the septum of the dividing cells where is addressed probably by its interaction with specific receptors positioned at the polar region on the pneumococcal surface [110]. The protein presents a limited cell-wall hydrolytic efficiency *in vitro*, probably due to a very strict substrate range that limits hydrolysis only to certain bonds specifically located in the cell poles linking together the daughter cells [110,113]. In addition, it has been reported its direct contribution in biofilm formation [114] and nasal colonization of pneumococci [115,116], and it has been shown that immunization of mice with LytB induces protective antibodies in a mouse sepsis model [85], making this enzyme a good candidate for the development of improved vaccines against *S. pneumoniae*.

The full-length LytB hydrolase displays a modular organization that differs from most CBPs as the functional module is located at the C-terminal position. It comprises a cleavable N-terminal signal peptide 23 residues long followed by a CBM (LytB_CBM_) and by a C-terminal catalytic module (LytB_CAT_) (Figure 2) [110,111]. The LytB_CBM_ contains between 12 and 18 repetitions depending on the [117] strain, the length of each varying from 17 to 23 residues. In the case of R6, the CBM is formed by 18 standard CBRs, with the last 8 configuring an alternance of 21-aa and 23-aa sequences in pairs [110]. Remarkably, several CBRs contain the GYMA motifs that have been shown to configure non-canonical binding sites as described above.

**Figure 7:**
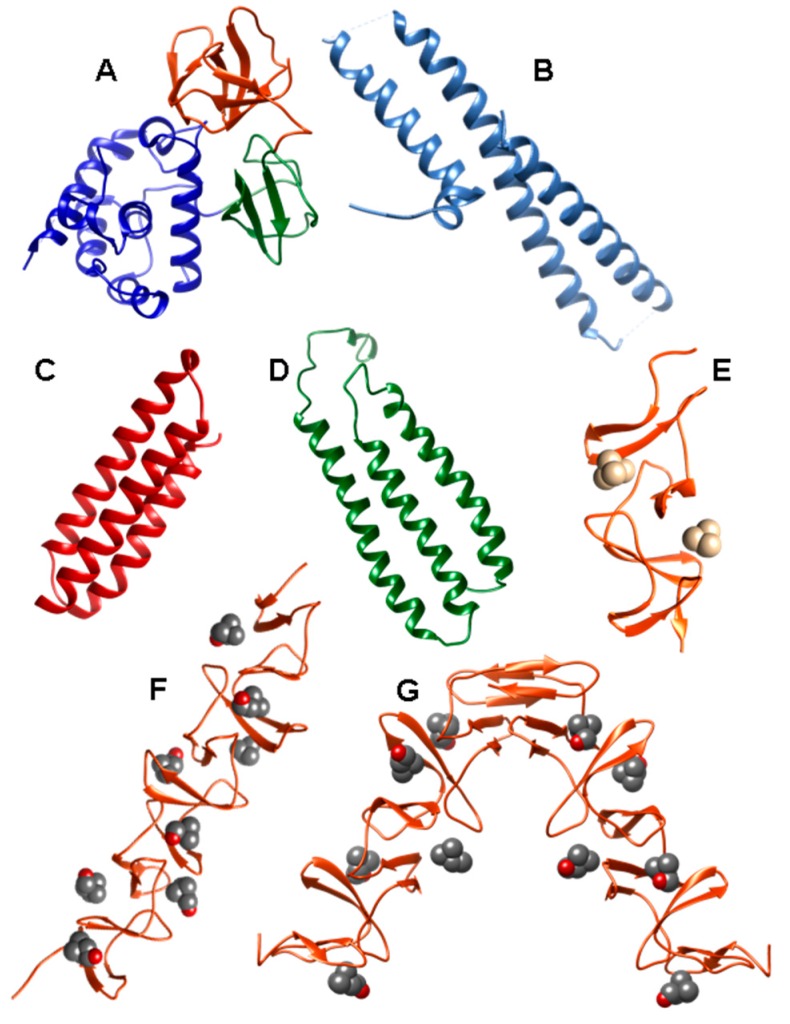
Three-dimensional structures of isolated domains from CBPs. (**a**) Functional module of LytB *N*-acetylglucosaminidase (PDB code: 4Q2W) (LytB_CAT_) depicting the SH3b, WW and GH73 domains in orange, green and blue respectively; (**b**) Lactoferrin-binding domain of PspA (PDB code: 2PMS); (**c**) CbpAN domain of PspC (PDB code: 2M6U); (**d**) R2 domain of PspC (PDB code: 1W9R); (**e**) Choline-binding module of CbpM (PDB code: 3HIA) containing *N*,*N*,*N*,*N*-tetramethylammonium in the binding sites; (**f**) Choline-binding module of CbpM (PDB code: 4CNL) bound to choline; (**g**) CBM module of prophage LytA (SPH_0121) (PDB code: 4IWT) in the presence of choline.

The catalytic module of LytB (LytB_CAT_) (PDB code 4Q2W) is connected to LytB_CBM_ by a 12-aa linker. It consists of three structurally independent domains (SH3b, WW and GH73) configuring a “T-shaped” pocket that accommodates a putative tetrasaccharide-pentapeptide substrate of the PG [118] (Figure 7A). SH3b and WW segments form two all-β structural modules, involved in specifically recognizing the bridging peptide structures of the cell wall PG. The GH73 module contains six α-helices and harbors the active site, including the catalytic residue Glu501 64, and three additional aromatic amino acids (Phe580, Tyr633 and Trp639) that create a hydrophobic environment for catalysis. All three modules are necessary for its optimal activity towards PG hydrolysis and for pneumococcal adhesion to respiratory epithelial cells [118].

### 2.3. LytC Lysozyme

LytC is an autolytic lysozyme that cleaves the β-(1→4)-glycosydic bond between the *N*-acetylmuramoyl-*N*-glucosaminyl residues that form the polysaccharide chain of the bacterial cell wall [119]. Together with LytA amidase, they represent the only two autolysins unequivocally identified in pneumococcus so far. The protein contains a leader peptide (33 residues) that directs the mature enzyme to the outer surface, where it is distributed all around the cell envelope although with a higher presence on the poles and septum of the division cells [80,119].

LytC has an optimal reaction temperature at 30 °C, suggesting that it might be active in the highly ventilated upper respiratory tract [119], and in fact, loss of function of LytC significantly reduces the nasopharyngeal colonization of rats [115]. The physiological role of this CBP also involves its participation in biofilm formation [114], but major attention has been centered on its noteworthy participation in cellular fratricide [120], a process that is directly related to natural transformation, increasing the rate of horizontal gene transfer within and between co-cultivated related species followed by homologous recombination [121,122,123,124]. In this manner, pneumococci get access to a common gene pool that confers them the capability to adapt more rapidly to the stress imposed by vaccination or the use of antibiotics [115,121], but also the virulence factors discharged by the lysed cells promote host invasion by the competent cells.

It has been shown that the relatively high concentration of LytC present in the medium at exponential growth phase of non-competent pneumococcal cultures is not harmful to the cells [94]. However, during the competent state, the CbpD murein hydrolase (see below) would induce the cleavage of PG stem-peptide, thus facilitating the hydrolysis of the non-cross-linked PG chains by LytC, inactive until this moment [80,94]. The damage induced by the combined action of both enzymes causes disruption of the cytoplasmic membrane followed by the release of intracellular LytA, and then extensive hydrolysis takes place.

In this regard, it has been claimed that the activity of LytC can be inhibited both *in vivo* and *in vitro* by CbpF, probably due to specific interactions between CbpF and peptidoglycan and preventing access of the lysozyme to its substrate [125] (see below).

Biochemical analysis has shown that LytC is a polydisperse protein in solution that monomerizes upon binding of choline [126]. The protein presents an overall hook-shaped conformation with an N-terminal CBM built by 11 CBRs (named p1–p11) distributed over two domains (NI and NII) plus a C-terminal catalytic module [80,119] (PDB codes: 2WW5, 2WWC and 2WWD) (Figure 5B). The NI domain contains the first nine CBRs (p1–p9) arranged in the usual solenoid conformation and including a non-canonical GYMA site at the end of the third repeat. They are involved in the binding of 7 choline residues, thus inducing the folding and stabilization of the protein [82]. On the other hand, the NII domain is formed by two modified repeats (p10 and p11), each folded as an antiparallel three-stranded β-sheet. This domain is not involved in choline binding but extensively interacts with the catalytic module and is oriented towards the active site. Despite its length and high number of choline binding sites, the LytC CBM is only marginally stable even in the presence of choline, staying unfolded at 37 °C and suggesting a molecular explanation for the absence of enzymatic activity at this temperature [82].

The functional module of LytC is formed by a single structural domain that folds into an irregular (β/α)_5_β_3_ barrel belonging to the glycosyl hydrolase GH25 family and containing the two acidic residues (Asp273 and Glu365) characteristic of lysozymes in the active site [80]. Notably, this configuration around the active site, that is oriented to the CBM instead of being fully exposed, imposes limitations on accessibility and hydrolysis of PG, which is degraded only when pre-processed by other hydrolases such as CbpD [80]. That is, the interaction of LytC with the substrate triggers the opening of the active site by a modular arrangement in which the NII domain plays a critical role [80]. In support of this, it has been shown that the activity of LytC on cell walls increases up to 130% when the substrate is pretreated with small amounts of LytA, providing a partial degradation of the peptide stem of PG [80].

### 2.4. CbpD Amidase/Endopeptidase

CbpD is a murein hydrolase [127] that is exclusively expressed during competence [128] and is secreted to the extracellular milieu via the Sec pathway [94]. When mixed cultures of attacker and target cells are grown on blood agar plates, CbpD assists in fratricide together with LytA, LytC and CibAB [128]. Instead, it plays a key role in liquid cultures [129], not by causing a substantial cell lysis by itself, but by binding to the poles and the division zone of the target cells [94] and digesting the peptide cross-linkers of the PG molecule, thus providing a more accessible substrate for LytC and thereby triggering cell wall hydrolysis [80,94].

CbpD is localized in the cell division region [127]. It comprises an N-terminal functional module containing a CHAP (cysteine, histidine-dependent amidohydrolases/peptidases) domain followed by two SH3b domains, plus a C-terminal CBM comprising four CBRs. Although the enzymatic specificity of CbpD is not already known, the presence of the CHAP domain, which has been proven in other systems to present *N*-acetylmuramoyl-l-Ala amidase or endopeptidase activities [130,131] has let to propose one of these actions for this protein [127]. The SH3b modules recognize the PG, while the CBM establishes interactions with the TAs [127]. This dual binding of the CbpD to TA and PG is required for efficient killing of non-competent cells during fratricide [127].

### 2.5. Choline-Binding Protein CbpF

CbpF is one of the most abundant proteins present in the pneumococcal cell wall. It does not possess any catalytic activity but acts as a regulator of the function of the LytC lysozyme [125]. This inhibitory effect is also displayed *in vitro* on CPL1 and CPL7 phage lysozymes, which share the same catalytic specificity as LytC, but not on other cell-wall hydrolases such as the LytA amidase or the LytB glucosaminidase [125].

The CbpF protein is expressed with a 28-aa signal sequence followed by two well-defined modules, and it is strictly composed of a combination of canonical and non-canonical CBRs [125,132]. The three dimensional crystal structure of the mature CbpF in complex with choline [125] (PDB codes: 2V04, 2V05, 2VYU, 2X8M, 2X8O, 2X8P) reveals an elongated shape for the protein (Figure 5C). The N-terminal module acquires a disc-shape conformation with four non-canonical CBR-like sequences (named dp1 to dp4) highly modified both by additional amino acids and by mutations on different positions of the consensus motif. No choline molecules are found in this region in the crystal structure of the protein [125], probably due to the relatively big size of the pocket between the dp hairpins and the presence of only two aromatic residues instead of the usual three, although voluminous choline analogs that behave as CBP inhibitors, such as ipratropium, nicely fit in these sites [133]. In any case, experimental data suggest that the N-terminal module of CbpF interacts with the cell-wall PG preventing the access of LytC or other lysozymes to the scissile bonds in the substrate [125]. These non-choline-binding, CBR-like sequences in the N-terminal moiety add a new evidence on the versatility of the *CW_binding_1* motif family, to which the CBRs belong, and that contains many members involved in glucan binding in streptococci and other bacterial species [134,135].

The CBM is able to bind 7 choline molecules and presents the superhelical fold characteristic of all CBPs. It is arranged into two regions: the CI domain, adjacent to the N-terminal module and made up of two modified CBRs (dp5 and dp6), and the CII domain containing five standard CBRs (termed p1–p5) plus a carboxy-terminal tail of 16 amino acids [125].

### 2.6. Pneumococcal Surface Protein A (PspA)

PspA is a highly variable CBP regarding antigenicity and molecular weight that is localized in the membrane [63,136,137,138,139]. It is present in practically all clinically important serotypes [136] and appears essential for full pneumococcal virulence [140,141], as it interferes with complement activation [142,143,144], and binds lactoferrin (an iron transporter that is involved in innate immunity) both in its *apo* and *holo* forms, thus protecting pneumococci from the killing by this polypeptide [145,146,147,148].

PspA is a highly immunogenic protein [149], and active and passive immunity to this protein can protect mice against pneumococcal infection [136,150]. Moreover, although it is a polymorphic protein among strains, it is sufficiently cross-reactive immunologically [137,151], so that immunization with a single PspA can protect against strains of highly diverse serotypes and in fact it has been widely proposed to be used in the formulation of human vaccines to extend the breadth of protection [86,87,88,89].

Structural characterization of PspA reveals that it is built from a highly helical functional module followed by a CBM and a 17-aa hydrophobic tail at the C-terminus [152]. The N-terminal domain contains a very elongated helical antiparallel coiled-coil structure that may extend beyond the capsule [153,154]. This domain has been shown to be essential for full pneumococcal infectivity [151], and constitutes the immunologically protective region of the protein [150,155]. The lactoferrin binding region of the protein has been localized within residues 167–288 of this first α-helical domain [156], is negatively charged and consists on one short helix followed by three helical segments in antiparallel conformation connected by mobile loops [147] (PDB code: 2PMS) (Figure 7B). It has been shown that this moiety is involved in electrostatic binding with the cationic lactoferrin, thus preventing the latter to reach the cell membrane [147]. Finally, a proline-rich sequence connects the functional domain with the CBM, composed of ten highly conserved CBRs.

### 2.7. Pneumococcal Surface Protein C (PspC)

The major adhesin of pneumococcus is PspC, (CbpA, SpsA, PbcA) [157,158]. This multifunctional protein is an important virulence factor that plays a key role in pneumococcal infection. PspC contributes to nasopharynx and lung colonization [157,159], promoting invasion [160,161] by specific binding to the human polymeric immunoglobulin receptor (hpIgR), an integral membrane protein required for transcytosis of IgA and IgM across the mucosal epithelial cells, and more specifically to the secretory component (SC) [158,162,163]. It has been proposed that this interaction might be managed by the bacteria to exploit the host cell polymeric immunoglobulin transcytosis machinery to promote its translocation across the mucosal barrier [162]. Moreover, PspC interacts directly with a laminin-specific integrin-receptor ubiquitously expressed on vascular endothelial cells, contributing to invasive diseases, including pneumococcal meningitis [164].

To evade the host immune response pneumococci have developed many strategies that include the participation of this protein. PspC can interact with the secretory IgA (sIgA) [158], which is the predominant antibody isotype in mucosal secretions, playing an important role in mucosal immunity [165], so that this interaction may prevent the clearance of bacteria. The alternative complement pathway activation can be prevented by PspC through its ability to bind complement factor H (FH) in a specific way and inhibiting the C3 convertase activity [144,166,167,168,169,170]. In addition, the classical pathway activation may be avoided through its binding to C4BP [171]. Finally, PspC has been identified as a human vitronectin binding protein, thus preventing deposition of the terminal complement complex (TCC) and subsequent bacterial lysis [172]. All this efficient interplay allows the bacteria to evade the host immune attack, preventing the extensive complement deposition in combination with its paralogous PspA [64,144,173]. As in the case of PspA, PspC has been proved as an immunogen in the mouse model and to be a good candidate for anti-pneumococcal vaccines [64,86,87].

Analysis of the nucleotide sequence of pneumococcus *pspC* locus reveals a high allelic variation from strains [64,174]. Based on multiple sequence alignments PspC proteins have been divided into 11 different groups [174]. All variants show common traits: an N-terminal, 37-aa leader peptide, followed by a long multifunctional module, a proline-rich region, and a C-terminal binding module, responsible for the attachment to the cell surface. Although the configuration of the entire protein is not known, the three-dimensional structure of some of its domains has been elucidated for the TIGR4 protein. Following the signal peptide, the CbpAN domain (amino acids 68–147 in the TIGR4 strain) consists of a three α-helical antiparallel bundle (Figure 7C) that binds specific, single FH domains [175] (PDB codes: 2M6U and 4K12) as demonstrated before [176,177,178]. The C-terminal part of the first helix and most of the second one constitute the interaction interface in which the insertion of Tyr90 from helix 1 into a “hydrophobic lock”, that is only present in certain FH domains, is key for binding and for determining the host specificity of the pneumococcal protein [175,179].

Next in sequence, the two “repeated”, multifunctional domains R1 and R2, of very similar sequence, are involved in hpIgR recognition [163]. The 3D structure of the R2 domain has been elucidated [180] (PDB code: 1W9R) (Figure 7D), and the structure of R1 modeled on the basis of their highly similar sequence (nearly 80% identity). These domains exhibit a cluster of 12 imperfect copies of the leucine zipper motif adopting an unusual three-α-helix, flat raft-like structure. The loop between helix 1 and helix 2 of each R domain contains the hexapeptide motif (Y/R)RNYPT, termed “tyrosine fork”, which has a fundamental role in the binding to SC and sIgA [162,163,180]. On the other hand, PspC binds the laminin receptor in an event that might modulate the access to the central nervous system [164]. The primary binding site for this binding is localized in the surface-exposed loop between helix 2 and 3 of R2 domain, displaying the highly conserved recognition sequence EPRNEEK [164]. Furthermore, both R domains have been shown to be required for the efficient interaction of *S. pneumoniae* with vitronectin, thus inhibiting complement function [172].

Finally, the major difference between the PspC groups is established by the C-terminal region, which is preceded by a proline-rich region: classic PspC polypeptides (groups 1–6) contain a CBM with 4–13 CBRs depending on the strain (8 in TIGR4) [64,174], followed by a hydrophobic stretch of 18 amino acids; on the other hand, the PspC-like proteins (Hic proteins, groups 7–11) display, instead of the CBM, the typical LPXTG motif [64,174] that is characteristic for most surface proteins in Gram-positive bacteria [181], and establishing a covalent interaction with the peptidoglycan.

### 2.8. Phosphorylcholine Esterase (Pce)

The TA phosphorylcholine esterase Pce (also known as CbpE and LytD) catalyzes the hydrolysis of about 20% of the total P-Cho residues from teichoic and lipoteichoic acids attached to the bacterial envelope [182,183,184]. Thus, it is involved in the distribution and restructuration of the content of choline in the cell wall, and in modulating indirectly the activity of other CBPs and the pathogen-host interactions [185]. In this sense, it has been postulated that upon removal of selected P-Cho residues, recognition of bacteria by host C-reactive protein (CRP) is diminished, providing the pathogen a mechanism to avoid the defense mechanism of the host [47].

The *in vivo* activity of Pce is associated to nasopharynx colonization and adherence to human epithelial cells [115]. Moreover, it plays a role in the recruitment of host proteases involved in cleavage of the extracellular matrix, as it interacts with human plasminogen, facilitating the dissemination through the host tissues [186]. In this sense, the inactivation of Pce induces a change in the formation of the colonies morphology, and also a significant increase in virulence when tested in mouse [183].

The *pce* gene encodes for a pre-protein with an N-terminal, 25-aa-long peptide sequence. After cleavage, the mature protein is secreted by the components of the general secretory pathway [183]. The enzyme is a metalloprotease that comprises a globular N-terminal catalytic module containing a zinc binuclear center followed by a short linker region that connects (and strongly interacts) with a very elongated C-terminal CBM plus a C-terminal tail of 85 residues [183,184,185]. Sedimentation velocity experiments evidence that Pce enzyme is a monomer both free and bound to choline [187]. Besides, a very restricted disposition of its modules is found both in solution and in the crystal structure that could also determine the release of only specific P-Cho terminal residues [185,187].

The structure of a recombinant protein lacking the C-terminal tail has been elucidated by X-ray crystallography in complex with the reaction product phosphocholine and choline analogues [185] (PDB code: 2BIB) (Figure 5D). Moreover, the struture of the isolated catalytic domain has also been described [188] (PDB code: 1WRA). According to these reports, the N-terminal catalytic module is formed by a single globular domain structurally constituted by an αβ/βα sandwich which follows the metallo-β-lactamase fold, although major structural and functional features makes it unique among this group of proteins [185,188]. It can be divided into two near-equivalent regions, each consisting of an anti-parallel β-sheet followed by three α,β-motifs. The active site contains two Zn^2+^ ions firmly attached to the protein at the bottom of a deep hole, stabilizing the catalytic module through a complex metal-ligand coordination network and resulting essential for the catalysis [185,189]. The major interaction between P-Cho and the enzyme occurs between the phosphate group of P-Cho and the binuclear center. In this sense, the configuration of the active site determines that only the P-Cho residues located at the end of the TA chains are accessible, thus releasing only those that are relevant for cell-cell interaction and providing a mechanism of escaping the attack by the host defense system [185]. On the other hand, the C-terminal CBM comprises 10 CBRs (termed p1–p10) of about 20 amino acids strictly arranged into a typical left-handed superhelical fold [185]. Finally, although the next 21-aa stretch following the last CBR (residues 517–534) does not bear any sequence homology, this region folds into the same hairpin structure as CBRs and, in fact, a non canonical binding site is constituted by two aromatic residues from this region (Tyr524 and Trp532) and two more from the last CBR (Trp 498 and Trp505) [185]. The role of the 85-aa C-terminal tag is still unknown.

### 2.9. Other Choline Binding Proteins

The *bp*I gene SP0069 in *S. pneumoniae* is present in TIGR4, but not in other studied strains such as R6, Hu19A_6, CGSP14 or the multiresistant Sanger 23F^−1^ [43]. It codes for a protein, CbpI, which has been crystallized, although the structure has not been elucidated yet [190]. It presents an N-terminal sequence without homology with other previously identified CBPs, and a CBM in the C-terminus with 5 CBRs [58]. It has been proposed to behave as a putative adhesin [191] that is involved in colonization of the nasopharynx [115]. Moreover, it has been shown to interact with elastin and the protein C reactive [58], but it is not a virulence factor in sepsis [115].

The *pcpA* gene codes for the PcpA (CbpN) protein [192], which is present in all the clinically relevant pneumococcus strains studied so far [58,192,193]. Its transcription is under the control of the manganese-dependent regulator PsaR, being expressed in log-phase growth cultures only at low manganese concentrations [193,194], whereas opposite effects are produced by nickel, zinc and cobalt divalent cations [195,196,197]. In this sense, it is predicted a lower level of expression in the nasopharynx and a higher level of expression in the lung and blood, and in fact, PcpA has been shown to be a virulence factor in a mouse model for lung and systemic infection [193,194,198], but is not involved in colonization [193,194]. Considerably, it elicits a "tissue site-specific" protection, being stronger against lung infection than sepsis in murine models, whereas it fails to immunize against colonization [193]. PcpA is a member of the LRR_TP_ subfamily of proteins, mostly involved in surface adherence or aggregation, as it contains several leucine-rich repeats in its N-terminal module [192,193], and it has been classified as a putative pneumococcal adhesin [38,56,58]. On the other hand, the C-terminus presents a CBM built up by 6–11 identical CBRs of 20 amino acids plus a tail of 19 amino acids [192].

CbpG is a multifunctional protein with proteolytic and adhesin function [115,199]. Together with PspA, they are currently the only proteins directly implicated in pneumococcal virulence in sepsis [115]. Sequence analysis of *cbp*G from a variety of laboratory and clinical isolates revealed two allelic variants. One third of the isolates, including R6 and D39 strains, contains a truncated form resulting from a stop codon prior to the C-terminal CBM [199]. Besides, both the truncated and the full-length variants have been isolated from invasive disease, suggesting that the truncation of CbpG does not appear to eliminate disease potential. On the other side, removal of the entire gene or truncation of the CBM decreases adherence in more than a 70%, indicating that the function of CbpG in adherence requires the CBM. Finally, it is interesting to note that CbpG vaccination has been shown to protect against colonization and systemic infection in a mouse model [199]. All CbpG variants present an N-terminal module displaying sequence similarity with trypsin-like serine proteases [115,199]. When present, the CBM in CbpG contains 3 CBRs, constituting the shortest CBM described so far [42]. In any case, proteolytic activity does not depend on whether the enzyme contains the CBM or not.

CbpM is encoded by the *spr*1274 gene in R6, being the TIGR4 SP1417 locus a pseudo-gene [58]. It has been shown that it interacts with CRP, and that it also binds elastin weakly [58]. With only 129 residues, it is the smaller CBP identified up to now and contains an N-terminal functional module, with adhesin function [58], followed by a CBM with three CBRs plus a C-terminal tail of similar sequence to that of CbpF [200]. The crystal structure of the CBM has been solved (PDB code: 3HIA) (Figure 7E) and reflects the configuration of two choline-binding sites [200]. The crystal contains three molecules in the asymmetric unit, forming a pseudo-trimer, although other data suggest that both CbpM and its isolated CBM are mainly present as monomers and dimers in solution [200].

Little information is available for other CBPs. The CbpL protein (encoded by *spr*0583 and SP0667 in the R6 and TIGR4 strains, respectively) presents two small domains referred to as Excalibur (Exc) and Lipoprot (LP). It has been classified as an adhesin, and it interacts with collagens, elastin and CRP [58], but its function remains poorly characterized. The three-dimensional structure of the CBM has been released (PDB code: 4CNL) [201] (Figure 7F) and displays the usual β-solenoid configuration. On the other hand, the protein coded by the orthologous genes SP0391 from TIGR4 and SP0351 from R6 has been renamed as CbpK [58], instead of CbpF and PcpC as previously termed [42,115,125,202,203]. Finally, CbpJ is coded by *sp*0378 in the strains TIGR4, CGSP14 and Sanger 23F^−1^, whereas it is absent in R6 and Hu19A_6 [42]. It is a putative adhesin that interacts with the host CRP [58], whereas it does not seem to be a virulence factor, nor involved in nasopharynx colonization [115].

## 3. Phage-Encoded CBPs

Endolysins (or lysins) are highly evolved bacteriophage encoded enzymes that specifically hydrolyze peptidoglycan bonds, disrupting the integrity of the bacterial cell wall, and allowing the releasing of the phage progeny particles [204]. Pneumococcal endolysins lack signal sequences, and the translocation through the cytoplasmic membrane to attack their substrate in the peptidoglycan is carried out by a holin system [48].

All the endolysins identified up to now from pneumococcal phages are characterized by a two-module structure, in which the N-terminal is responsible for the catalytic activity of the enzyme, whereas the C-terminal module allows the binding to the targeted cell wall [48]. With the only exception of CPL7 (coded by Cp-7 phage), the cell-wall binding modules of endolysins consist of a typical CBM, so that presence of choline in the TA is required for optimal substrate recognition. Only endolysins with amidase or lysozyme activities have been described so far. Examples of phage amidases are Pal (from Dp-1 phage), Ejl (from EJ-1), Hbl (from HB-3), Mml (from MM1), and LytA-VOl (from VO1) [48]. These amidases display sequence homology with the host LytA autolysin. Interestingly, the Pal amidase seems to be a natural chimeric enzyme of intergeneric origin, with its N-terminal module highly similar to that of a murein hydrolase from phage BK5-T that infects *Lactococcus lactis*, and the C-terminal module displaying a 64.6% identity with the LytA amidase [205]. On the other hand, the lysozyme group includes the CPL1, CPL7 and CPL9 enzymes, coded by Cp-1, Cp-7 and Cp-9 phages, respectively [61]. They all exhibit a highly conserved N-terminal catalytic module belonging to the glycosyl hydrolase family 25 (GH-25). CPL1 and CPL9 are nearly identical proteins, whereas the cell-wall binding module of CPL7 consists of three identical repeats of 48 residues with a completely different structure to the CBM, so that this endolysin displays a choline-independent activity [61].

To date, CPL1 is the only full-length phage CBP which 3D structure has been elucidated, both in free state and complexed with choline and cell wall PG analogues [81,206] (PDB codes: 1H09, 1OBA, 2IXU, 2IXV, 2J8F, 2J8G) (Figure 5E). It is folded in two well-defined modules connected by an acidic linker, and the protein dimerizes in solution through its C-terminal module in the presence of choline [187]. The catalytic module consists of an irregular (β/α)_5_β_3_ barrel with a long cleft that constitutes the substrate binding site. Two acidic residues (Asp-10 and Glu-94) are involved in classical lysozyme acid-base catalysis, and it is thought that the enzyme acts by a processive mechanism [206]. On the other hand, the CBM is made up of six CBRs (termed p1–p6), each forming a β-hairpin, plus a C-terminal tail of 16 amino acids. The first four repeats (p1–p4, CI domain) are arranged in a super-helical structure, whereas the other two (p5–p6) and the C-terminal tail fold as an antiparallel six-stranded β-sheet (CII domain) that establishes a strong interaction with the functional module that should restrict access to the substrate, although this constrain may be relieved in solution [187]. Despite having six CBRs highly similar to those of LytA [59] there are important differences among these two CBMs. Only two choline-binding sites, located at the interfaces of the three first repeats (p1–p2 and p2–p3), can be seen in the crystal structure of CPL, instead of 6 ligands for LytA [83]. Moreover, although both proteins acquire a similar “boomerang-like” shape upon dimerization through the CBM, it is the N-terminal region of the CPL1 module the one that is involved in protein-protein contacts, configuring a possible additional choline-binding site in the dimer interface [187].

Regarding three-dimensional structures of other phage-derived CBPs, only recently it has been reported the configuration of the CBM module of prophage LytA (SPH_0121) from the Hungary 19A-6 strain (ATCC 700673), of nearly identical sequence compared to host LytA autolysin [102]. As a consequence, it shares a very similar dimeric, boomerang-like shape, as well as choline-binding characteristics.

## 4. Actions to Control Pneumococcal Infections Based on CBPs

### 4.1. Enzybiotics

One therapeutic antipneumococcal strategy that has demonstrated an important efficiency involves the use of recombinant phage- or bacteria-encoded CBP enzymes to lyse the cell wall when externally added. Fischetti and coworkers first demonstrated that purified CPL1 was able to kill most serotypes of pneumococci both *in vivo* and *in vitro*, and first coined the term “enzybiotic” for designating these kind of enzymes [207]. Subsequently, different phage endolysins have been successfully tested *in vitro* and in several animal models, both alone or in combination with other antimicrobial agents, and they have been extensively reviewed in recent years [204,208,209,210,211] (Table 1). Currently, the term “enzybiotic” has been extended to all enzymes, regardless of their origin, exhibiting antibacterial and/or antifungal activity [212,213].

Pneumococcal phage CBP lysins act first by tight binding to the cell wall substrate through their CBM, and then digesting the peptidoglycan in localized areas, creating holes that lead to the death of the targeted bacteria. As these proteins are only active on microorganisms containing choline in their envelope, they have the advantage of interfering neither with the usual microbiota, nor with the mammalian tissues. The bactericidal activity of phage lysins has been characterized using a variety of animal models including of pneumonia, endocarditis and sepsis, with a focus on efficacy and host immune response (Table 1) [214,215,216,217,218,219,220]. Endolysins have been proven to be effective on preventing pharyngeal colonization [207,214], and it has been proposed that nasal sprays containing these enzymes could be an effective alternative to conventional antibiotics, helping to eliminate human reservoirs of the bacteria. Another interesting study shows, in a mouse model of otitis media, that 80% of mice colonized with *S. pneumoniae* naturally developed otitis media upon infection with influenza virus. Previous treatment of the mice with CPL1 lysin showed to be effective at preventing the development of otitis media in 100% of the cases [218]. Since around half of all children are colonized with *S. pneumoniae* [6], which is a leading cause of otitis media, the treatment of high-risk individuals during the influenza season to decolonize them could reduce secondary infections by these bacteria [218]. Special mention should be done to the work of Witzenrath and coworkers, as they demonstrate the therapeutic potential of CPL1 in mice with a well established and severe pneumonia, and even when animal developed bacteremia [220].

Synergic actions between different endolysins such as CPL1 and Pal have been described both *in vitro* and *in vivo* [216,221]. The positive interaction has been postulated to be due to an increased access of the enzymes to the respective cleavage sites or an enhanced destructive effect of a bidimensional simultaneous digestion of the peptidoglycan [221]. Besides, another synergy has also been observed *in vitro* or *in vivo* between endolysins and antibiotics, this combination being highly lethal against different pneumococcal strains, including an extremely penicillin-resistant strain [222,223,224].

A step forward in the field of enzybiotics is represented by the work of Rodriguez-Cerrato and coworkers, as they used a host-encoded lytic enzyme (LytA), and demonstrated its effectivity against a β-lactam-resistant *S. pneumoniae* both *in vitro* and in a peritonitis-sepsis mouse model, resulting even more effective than the widely used phage-encoded CPL1 lysozyme [225].

Like many other proteins, endolysins induce an immune response. Nevertheless, this results only in a modest reduction of their activity that does not prevent their use as antibacterial agents [215,216]. Besides, no signs of toxicity have been reported in mouse models [207,214,215,216,218].

So far, specific resistance mechanisms have not been described against CBP enzybiotics [214], and the probability of their development is presumably low, as the cell wall receptor for the pneumococcal lysin is choline [226], a molecule that is essential for pneumococcal viability. An additional concern is the pharmacokinetics of these polypeptides, with a half-life of about 20 min in the body, similarly to other foreign proteins delivered systemically to animals [215,225]. However, some authors have reported such a high therapeutic efficiency that one single dose could be enough to be therapeutically sufficient [215]. Otherwise, it may be necessary to modify the structure by protein engineering and/or change formulation characteristics of these CBPs to increase its stability and allow its systemic use for longer periods. In this sense, the Fischetti group has developed a CPL1 dimer with an engineered disulphide bridge linking the CBMs that shows a two-fold increase in antipneumococcal activity and about a 10-fold decrease in plasma clearance [227]. Moreover, thanks to their modular organization, novel chimeric CBP lysins are amenable to be designed in which the specificity and activity could be tailored for their therapeutic use [66,228].

### 4.2. Inhibition of CBPs

Given the high variety of roles carried out by CBPs, it turns out that specific inhibition of their functional modules by the use of small molecules would undoubtedly affect key aspects in pneumococcal vital cycle. However, the search for inhibitors for each individual CBP may be a laborious task, and the elucidation of the three-dimensional structure of functional modules will certainly be essential in the design of antimicrobials specifically tailored to sensible regions in each particular CBP. In any case, the extremely similar architecture and biochemical behavior of all CBMs studied so far suggest the alternative approach of employing a single molecule that binds to the choline-binding sites in the CBMs and prevents the attachment of most or all CBPs to their destinies in the cell surface. Taking into account the variety of CBP activities discussed above, the rationale is that the general inhibition of CBP function by specific molecules should bring deleterious consequences for pneumococcal viability and virulence, for instance: (*i*) a diminished adherence to the host tissues; (*ii*) reduced internalization; (*iii*) prevention of daughter cell separation upon division, leading to easily phagocytable long chains with diminished infectivity; (*iv*) a decreased LytA-promoted capsule shedding on response to host antibacterial peptides; (*v*) prevention of release of virulence factors; (*vi*) interference with pneumococcal “fratricide”; and (*vii*) a higher concentration of exposed choline residues in the surface (arising from general CBP elution from the cell wall as well as the inhibition of Pce phosphorylcholinesterase activity), favoring the activation of the complement system and the subsequent potentiation of immune response. In support of this hypothesis, it has been reported for instance that LytA-inactivated strains are less virulent [95,229,230,231]. On the other hand, caution should be observed as inhibition of particular CBPs may induce undesirable side effects, such as the increase of tolerance to antibiotics when LytA is inhibited [96,232] or the inability of cure of infection. Nevertheless, it is expected that the numerous detrimental effects arising from the overall collapse of CBP function counterbalance such drawbacks. Moreover, since a long family of proteins would be affected simultaneously, the rising of antimicrobial resistances by point mutations in individual CBPs should be more difficult to occur.

**Figure 8:**
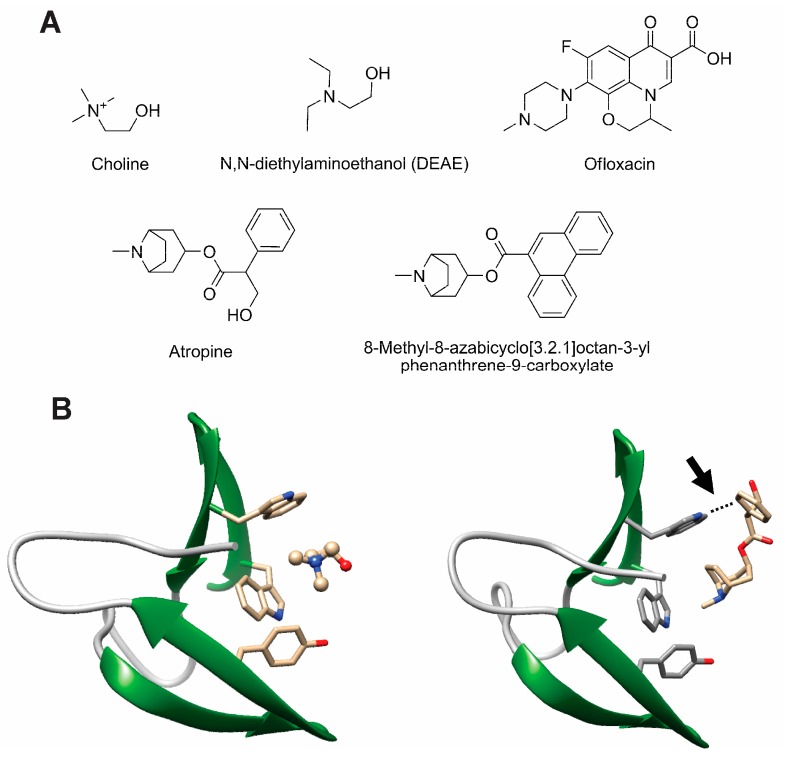
Structure of representative choline analogs that bind to CBPs. (**a**) Chemical formulae of representative compounds; (**b**) Comparison of the same choline-binding site in CbpF occupied by choline (**left**, PDB code: 2V04) or by atropine (**right**, PDB code: 2X8P); the arrow indicates the additional π-π interaction that provides high stability to CBP-EBA complexes.

Choline itself is the first candidate to be considered as CBP inhibitor, as it is long known that it interferes with the activity of CBPs *in vitro* and that the addition of this compound to liquid culture media hinders the activity of LytA [233,234], avoiding daughter cell separation and leading to the appearance of long cellular chains [234]. Promoting pneumococcal chain formation might constitute an effective antimicrobial approach as such chains should be more easily phagocytable and less infective. In addition, it has been revealed that *S. pneumoniae* avoids complement-mediated killing by minimizing its chain length in a process in which LytA might play a pivotal role [235]. However, while CBPs strongly attach to the multiple choline-containing TAs, they only show a weak affinity for choline in solution [82,84,105,236] so concentrations of choline in the tens of millimolar range are needed to affect their activity, which is therapeutically non-viable. This makes necessary the development of choline analogs that possesses a higher binding efficiency. In fact, CBMs display a broad ligand range [104,133,237,238,239] (see representative compounds in Figure 8A) in which the minimal requirement for binding is that of a tertiary amine with two ethyl substituents or a quaternary amine with three methyl substituents [237]. The choline-binding sites are able to accommodate longer substituents provided they are non-polar. A current biotechnological application of the C-LytA module is based on exploiting this affinity for choline analogues such as *N*,*N*-diethylaminoethanol (DEAE, Figure 8A) [237], leading to the development of single-step chromatographic immobilization and purification systems of fusion proteins containing this CBM [240,241,242,243,244].

An *in silico* docking search of a library of compounds led to the selection of several putative CBP inhibitors by Romero and coworkers [104]. Among these, fluoroquinolone antibiotics such as ofloxacin (Figure 8A) and analogs demonstrated a 10-fold higher *in vitro* efficiency compared to choline. Similar inhibition levels were also initially reported with other non-antibiotic compounds such as aromatic esters of bicyclic amines (EBAs) like atropine and ipratropium [238] (Figure 8A). However, EBAs do not behave as mere choline analogs since they do not induce chain formation *in vitro*, but they reduce and even arrest cell growth, decreasing bacterial viability in more than 90% [238], and showing therefore a strong therapeutical potential. Analysis of crystal complexes between CbpF and atropine points, as the major determinant for the stronger EBA binding compared to choline, to the simultaneous involvement of protein tryptophan residues in the binding site both with the ammonium group in the ligand (through cation-π forces) and with its aromatic moiety (involving T-shaped π-π stacking) [133] (Figure 8B). On the basis of these structural data, further screening of rationally designed chemical libraries of EBA derivatives for binding to the CBM from LytA (C-LytA protein) led to optimized compounds with a high content in aromatic rings (Figure 8A) that inhibit *in vitro* lytic activity of LytA with a 100-fold stronger efficiency than choline [239]. Moreover, these EBA derivatives arrest liquid pneumococcal cultures *in vitro* of both non-capsuled R6 and capsuled D39 strains, with minimal inhibitory concentrations (MIC) in the low micromolar range. These values are comparable to usual β-lactam, macrolide, and fluoroquinolone antibiotics [245]. Bactericidal results were also validated in an *in vivo* zebrafish embryo model of infection, reaching 98% survival at a 2 μm concentration [239].

**Figure 9:**
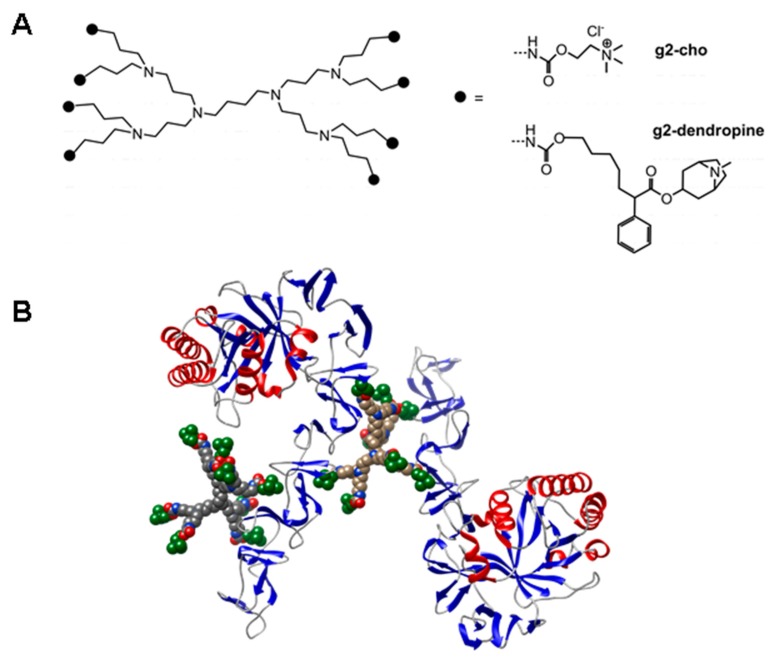
Dendrimeric nanoparticles as inhibitors of CBPs. (**a**) Structure of generation-2 poly(propylene imine) dendrimer functionalized with 8 molecules of choline (g2-cho) or atropine (g2-dendropine); (**b**) A model of the polyvalent interaction between a CBP such as LytB (in ribbon representation) and g2-cho dendrimers (in van der Waals representation). Terminal choline residues are colored green.

An alternative procedure is represented by a recent nanotechnological procedure that involves the use of a poly(propylene imine) dendrimer scaffold functionalized with several molecules of choline [246] or EBAs [239] (Figure 9).

The rationale of this approach is that these multivalent nanoparticles resemble the mosaic disposition of choline residues in the cell wall. Since a dendrimer contains several ligand copies on its surface, and the CBPs contain, in turn, several binding sites, multimolecular complexes are likely to be produced (Figure 9B), so that the binding affinity increases exponentially rather than linearly for entropic reasons [247]. As a result, choline dendrimers harboring between 4 and 64 choline residues inhibit the CBP activity *in vitro* 10,000-fold more efficiently than free choline, causing inhibition of daughter cell separation after division [246] and favoring phagocytosis by microglial cells [248]. On the other hand, a dendrimer containing only 8 atropine molecules and termed *g2-dendropine* (Figure 9) not only dramatically enhances its LytA inhibitory efficiency *in vitro* compared with atropine by 45,000-fold, with an IC_50_ in the nanomolar range, but also turns the non-lytic activity shown by the choline dendrimers into a lytic response, so that a micromolar concentration is sufficient to completely abolish growth of the R6 strain [239]. Similar results were also obtained with the highly pathogenic AR33118 (serotype 3) strain, a pneumococcal clinical isolate that displays resistance to levofloxacin [239]. These results demonstrate the antimicrobial potential of polydentate nanoparticles as a way of increasing the efficacy of antimicrobials.

## 5. Conclusions

The wide panoply of physiologically relevant processes in which CBPs are involved makes this family of proteins as very attractive subjects to open up new streamlined antipneumococcal alternatives to confront current and future antimicrobial resistance threats. The use of lytic CBPs as enzybiotics to treat infections has thoroughly demonstrated its potential and current efforts are now aimed to increase their *in vivo* stability and efficiency by the judicious use of protein engineering techniques as well as by the improvement of their formulation properties. On the other hand, development of drugs specifically targeting pneumococcal CBPs is still in its infancy but its importance might be boosted provided that biochemical, nanotechnological and computer screening efforts are jointly combined on the basis of the elucidation of new three-dimensional CBP structures.

## Figures and Tables

**Table 1:** *In vivo* assays of pneumococcal CBPs as enzybiotics on animal models.

CBP	Origin	Administration	Animal Model	Reference
CPL1	Cp-1 phage	Intravenous	Murine bacteremia	[215]
		Intravenous	Rat endocarditis	[217]
		Intranasal	Mice nasal colonization	[218]
		Intracisternal or intraparenteral	Rat meningitis	[219]
		Intraperitoneal	Mice severe pneumonia	[220]
		Oral	Mice nasal colonization	[207]
Pal	Dp-1 phage	Nasal and pharyngeal administration	Mice nasal colonization	[214]
CPL1 and Pal (separately or combined)	Cp-1 and Dp-1 phages	Intraparenteral	Murine sepsis	[216]
LytA	*Streptococcus pneumoniae*	Intraperitoneal	Mice peritonitis-sepsis	[225]
